# Development and validation of a CD4+/CD8+ ratio-based nomogram to predict plastic bronchitis in pediatric *Mycoplasma pneumoniae* pneumonia

**DOI:** 10.3389/fped.2025.1625206

**Published:** 2025-07-29

**Authors:** Di Lian, Chenye Lin, Xiangmei Dong, Jianxing Wei, Xueling Huang, Hongman Jiang, Qiuyu Tang

**Affiliations:** ^1^ Pulmonology Department, Fujian Children’s Hospital (Fujian Branch of Shanghai Children’s Medical Center), College of Clinical Medicine for Obstetrics & Gynecology and Pediatrics, Fujian Medical University, Fuzhou, Fujian, China; ^2^ Fuzhou KingMed for Clinical Laboratory Co., Ltd, Department of Laboratory Diagnosis, Fuzhou, Fujian, China; ^3^ College of Clinical Medicine for Obstetrics & Gynecology and Pediatrics, Fujian Medical University, Fuzhou, Fujian, China

**Keywords:** plastic bronchitis, *Mycoplasma pneumoniae*, pneumonia, nomogram, CD4+/CD8+ ratio, children, LASSO regression, bootstrap validation

## Abstract

**Introduction:**

Plastic bronchitis (PB) is a rare but severe complication of *Mycoplasma pneumoniae* pneumonia (MPP) in children. Early identification of PB is critical for timely intervention. This study aimed to develop and validate a nomogram incorporating the CD4+/CD8 + ratio to predict PB risk in children with MPP who underwent bronchoscopy.

**Methods:**

This single-center, retrospective cohort study was conducted at Fujian Children's Hospital, China, from January 2023 to December 2024. A total of 281 children hospitalized with MPP, including 39 patients who developed PB, were included. Key predictors for nomogram were selected out of 19 variables using least absolute shrinkage and selection operator regression, followed by multivariable logistic regression. Model performance was evaluated through discrimination, calibration, and decision curve analysis (DCA). Internal validation was performed using the Bootstrap method with 1,000 resamples.

**Results:**

The nomogram incorporated four independent predictors—fever duration, atelectasis, elevated D-dimer, and reduced CD4+/CD8 + ratio. It demonstrated strong discrimination (AUC = 0.83, 95% CI 0.77–0.90) and calibration (Hosmer-Lemeshow *P* = 0.303), with superior net benefit across risk thresholds of 0.1–0.7 by decision curve analysis, supporting its clinical utility for early risk stratification. Bootstrap validation confirmed robust performance with minimal overfitting.

**Discussion:**

CD4+/CD8 + ratio based nomogram provides a practical tool for predicting PB risk in children with MPP, which may facilitate early bronchoscopy leading to improved patient outcomes.

## 1 Introduction

*Mycoplasma pneumoniae* Pneumonia (MPP) is the leading cause of community-acquired pneumonia in children, accounting for 10%–40% of cases globally (1). Plastic bronchitis (PB) is a rare but critical complication of MPP that involves the formation of bronchial casts that obstruct airways. PB can lead to respiratory failure if not addressed promptly (2, 3).

Recent studies have reported an increasing incidence of PB in MPP cases, particularly among school-aged children, associated with prolonged fever, heightened inflammatory responses, and immune dysregulation (4, 5). The early identification of PB in MPP remains challenging due to its nonspecific clinical presentation and overlap with other respiratory conditions. Although bronchoscopy is the gold standard for diagnosis, its invasive nature limits routine use, highlighting the need for predictive tools based on accessible clinical and laboratory markers (6).

A number of predictive models have been proposed previously that included clinical and laboratory variables, such as fever duration, lactate dehydrogenase (LDH), D-dimer, and atelectasis, as as potential predictors of PB in RMPP (7). However, despite evidence of immune-mediated mechanisms in MPP severity, existing models overlook immune markers, such as the CD4+/CD8 + ratio (8). Immune dysregulation, particularly imbalances in T-cell subsets like the CD4+/CD8 + ratio, has been implicated in chronic inflammation and airway remodeling, which may predispose patients to the formation of bronchial casts in PB.Moreover, existing models lack validation in broader MPP cohorts and consensus on optimal predictors, limiting their generalizability. This study aimed to develop and validate a nomogram incorporating the CD4+/CD8 + ratio, alongside established clinical and laboratory variables, to predict PB risk in pediatric patients children with MPP.This model is specifically designed to identify high-risk individuals among MPP patients considered for bronchoscopy.

## 2 Methods

### 2.1 Study design and settings

This retrospective study was conducted at Fujian Children's Hospital, Fuzhou, China, from January 2023 to December 2024, to develop and validate a nomogram incorporating the CD4+/CD8 + ratio for predicting plastic bronchitis (PB) in children with MPP.

The study was approved by the Ethics Committee of Fujian Children's Hospital (Approval No. 2025ETKLRK04004), and informed consent requirement was waived due to the retrospective design, in accordance with the Declaration of Helsinki.

### 2.2 Inclusion and exclusion criteria

All patients with the diagnosis of MPP as per the Diagnostic and Treatment Guidelines for Child Pneumonia Caused by *Mycoplasma pneumoniae* (2023 Edition) (9) were included in the study if they fulfilled the bronchoscopy indications according to the Chinese Pediatric Flexible Bronchoscope Technique Guidelines (2018 Edition) (10).

A patient was diagnosed with MPP on the basis of (a) primary clinical manifestations of fever and cough, potentially accompanied by headache, nasal discharge, sore throat, or otalgia; (b) chest x-ray or CT showing thickened peribronchovascular textures, bronchial wall thickening, and possible ground-glass opacities, tree-in-bud sign, thickened interlobular septa, or reticular shadows; (c) single serum MP antibody titer ≥1:160 [Particle Agglutination (PA) method] or a four-fold increase in paired sera during the disease course; (d) positive MP-DNA or RNA detection. Diagnosis required clinical and imaging findings combined with either criterion (c) or (d).

Bronchoscopy indications included (a) pulmonary imaging anomalies, such as atelectasis or pleural changes, needing differential assessment; (b) determination and management of pathogens causing lung infections, with bronchoscopy conducted during hospitalization.

The exclusion criteria were as follows: (1) past or current tuberculosis, signs of tuberculous infection, repeated respiratory tract infections, persistent pulmonary conditions, asthma, primary or acquired immunodeficiency, hepatic or renal disorders, or cardiovascular conditions and (2) co-infection with other pathogens confirmed by laboratory testing.

### 2.3 Data collection

Data, including demographic information (age and sex), clinical features (fever duration,defined as the number of days from fever onset to hospital admission, wet rales, and wheezing), laboratory parameters (white blood cell count [WBC]; neutrophil percentage [N%]; erythrocyte sedimentation rate [ESR]; procalcitonin [PCT], C-reactive protein [CRP], lactate dehydrogenase [LDH], alanine aminotransferase [ALT], aspartate aminotransferase [AST], ferritin, fibrinogen, D-dimer, and interleukin-6 [IL-6] levels; and CD4+/CD8 + ratio, and imaging findings (atelectasis) were extracted from electronic medical records by two independent researchers to ensure accuracy. All data were collected at admission,prior to bronchoscopy, to ensure consistency in timing and clinical context for predictive modeling.

### 2.4 Pathogen detection via tNGS

*Mycoplasma pneumoniae* (MP) as the primary pathogen and exclusion of co-infections was based on targeted next-generation sequencing (tNGS) of bronchoalveolar lavage fluid (BALF) samples. The tNGS panel enabled detection of 153 pathogens, including bacteria, viruses, fungi, and atypical pathogens such as MP, *Chlamydia pneumoniae*, and *Legionella pneumophila*, as previously validated for comprehensive pathogen identification (11). The process involved nucleic acid extraction, library construction, high-throughput sequencing, and data analysis, conducted by Fuzhou Kingmed Diagnostics Company. BALF-tNGS results served as the diagnostic standard for MP infection, ensuring specificity in identifying PB cases associated with MP.

### 2.5 Statistical analysis

All statistical analyses were performed using R software (version 4.2.2, R Foundation for Statistical Computing, Vienna, Austria) and MSTATA software (https://www.mstata.com/). The normality of continuous variables was assessed using the Shapiro–Wilk test. Continuous variables were expressed as mean ± standard deviation (SD) for normally distributed data (e.g., WBC, N%, ESR etc.) or median [interquartile range (IQR)] for non-normally distributed data (e.g., fever duration, D-dimer etc.) ategorical variables were presented as frequencies and percentages. Differences between the PB and non-PB groups were assessed using the *t*-test for normally distributed continuous variables, the Mann–Whitney *U*-test for non-normally distributed continuous variables, and the chi-square test for categorical variables, with a significance threshold of *P* < 0.05.

Variable selection was conducted using least absolute shrinkage and selection operator (LASSO) regression to identify key predictors of PB from candidate variables. LASSO regression was performed with a penalty parameter (*λ*) selected via tenfold cross-validation, minimizing the binomial deviance. Variables with non-zero coefficients at the optimal *λ* (0.054) were selected and incorporated into a multivariable logistic regression model to construct a nomogram for predicting PB risk. Model performance was evaluated as follows: (1) discrimination was assessed by the area under the receiver operating characteristic (ROC) curve (AUC); (2) calibration was evaluated using a calibration curve and the Hosmer–Lemeshow test; (3) clinical utility was assessed via decision curve analysis (DCA); and (4) internal validation was performed using the bootstrap method with 1,000 resamples, adjusting for optimism in the C-index to quantify overfitting and ensure model robustness. All statistical tests were two-sided, and *P*-values of < 0.05 were considered statistically significant.

## 3 Results

**Figure 1:**
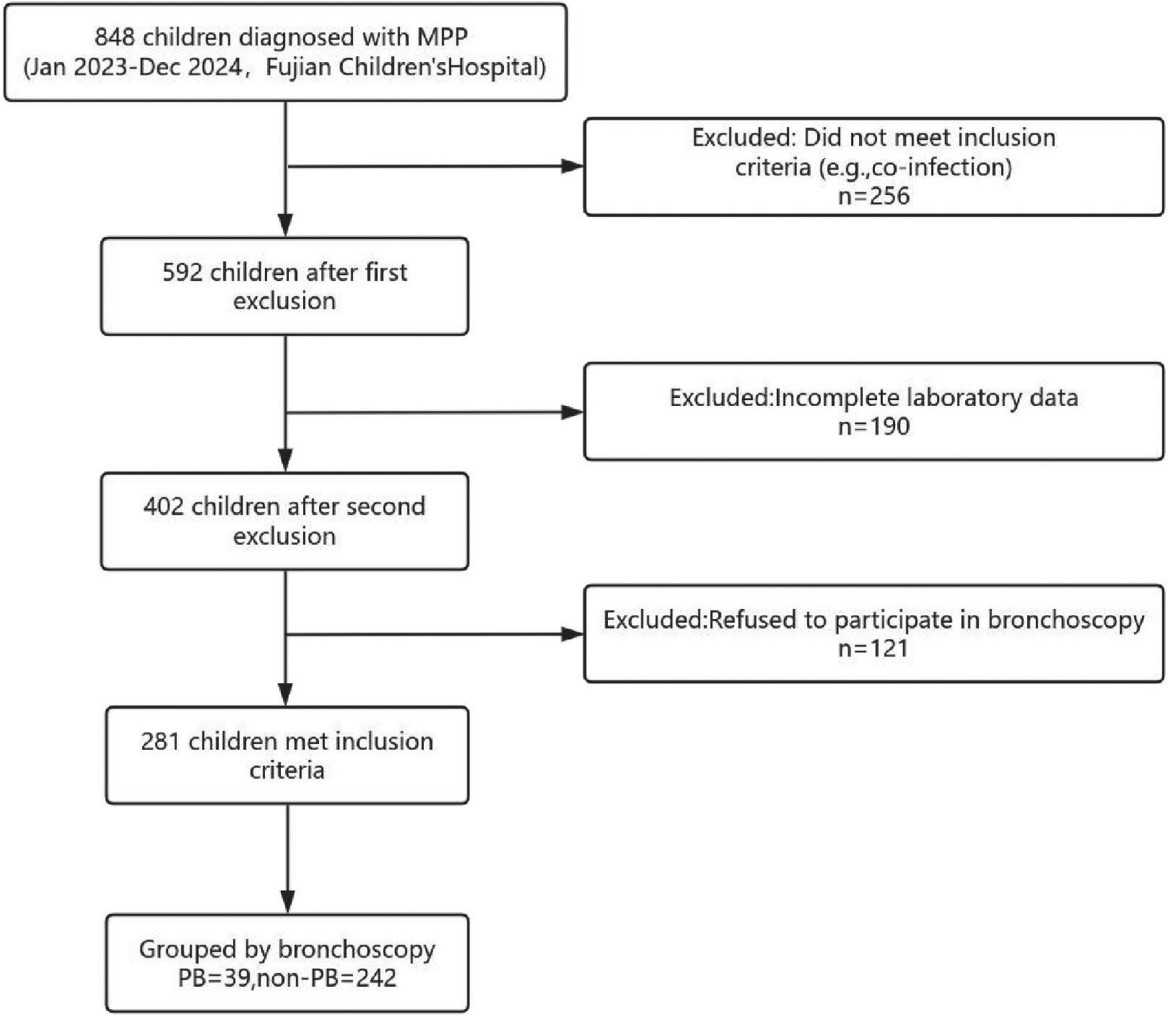
Flowchart of the participant inclusion process.

Total 848 patients were diagnosed with Mycoplasma pneumoniae pneumonia (MPP) during the study period at Fujian Children's Hospital. However, 567 patients were excluded due to incomplete laboratory data (e.g., missing CD4+/CD8 + ratio, *n* = 190), refusal to undergo bronchoscopy (*n* = 121), or not meeting inclusion criteria (*n* = 256). The 256 excluded patients included 2 with past tuberculosis infection, 32 with recurrent respiratory infections or chronic lung diseases (e.g., bronchiectasis), 183 with co-infections confirmed by targeted next-generation sequencing (tNGS), 28 with bronchial asthma on inhaled corticosteroid treatment, 9 with immune diseases, and 28 with liver, kidney, or cardiovascular diseases (e.g., patent ductus arteriosus), with some having overlapping conditions. Hence, total 281 pediatric patients with MPP were included. Out of these 281 patients 39 (13.88%) patients had confirmed plastic bronchitis (PB) on bronchoscopy (Figure 1).

### 3.1 Comparison of clinical characteristics and laboratory findings

The baseline characteristics and laboratory findings of the study population are summarized in Table 1. No significant differences were observed between the non-PB and PB groups in terms of age (*P* = 0.787), sex distribution (*P* = 0.242), the prevalence of wheezing (*P* = 0.167), or most of the laboratory parameters including WBC, N%, PCT, ESR, CRP, ALT, AST, fibrinogen) (Table 1). However, the PB group exhibited a significantly longer fever duration (*P* < 0.001), higher incidence of wet rales (*P* = 0.035), and atelectasis (*P* < 0.001) compared to the non-PB group. Laboratory findings revealed that the PB group had significantly higher levels of lactate dehydrogenase (LDH), D-dimer, and interleukin-6 (IL-6), alongside lower CD4+/CD8 + ratio and ferritin levels (all *P* < 0.05). These findings indicate that the PB group presents a distinct clinical and immunological profile compared to the non-PB group, suggesting specific pathophysiological pathways involved in PB development.

**Table 1:** Comparison of baseline characteristics and laboratory parameters between the Non-plastic bronchitis (Non-PB) and plastic bronchitis (PB) groups.

Variables	Non-PB (*n* = 242)	PB (*n* = 39)	Statistic	*P-*value
Sex, *n* (%)			*χ*^2^ = 1.37	0.242
Male	106 (43.80)	21 (53.85)		
Female	136 (56.20)	18 (46.15)		
Wet rales, *n* (%)			χ^2^ = 4.45	0.035
No	70 (28.93)	5 (12.82)		
Yes	172 (71.07)	34 (87.18)		
Wheezing, *n* (%)			χ^2^ = 1.91	0.167
No	202 (83.47)	29 (74.36)		
Yes	40 (16.53)	10 (25.64)		
Atelectasis, *n* (%)			χ^2^ = 30.74	<.001
No	221 (91.32)	23 (58.97)		
Yes	21 (8.68)	16 (41.03)		
WBC (×10^9^/L), Mean ± SD	8.27 ± 3.36	7.15 ± 3.93	t = 1.88	0.061
N%, Mean ± SD	60.76 ± 14.57	60.81 ± 22.37	t = −0.01	0.990
ESR (mm/h), Mean ± SD	51.24 ± 22.39	48.28 ± 23.55	t = 0.76	0.447
Age, M (Q₁, Q₃)	6.00 (5.00, 8.00)	6.00 (5.00, 8.00)	Z = −0.27	0.787
Fever duration, M (Q₁, Q₃)	6.69 (4.00, 8.65)	8.10 (7.00, 10.00)	Z = −3.78	<.001
PCT (ng/ml), M (Q₁, Q₃)	0.38 (0.28, 0.50)	0.40 (0.31, 0.59)	Z = −0.92	0.355
CRP (mg/L), M (Q₁, Q₃)	21.74 (16.27, 30.38)	22.39 (17.63, 31.47)	Z = −0.45	0.651
LDH (U/L), M (Q₁, Q₃)	323.30 (241.25, 481.00)	498.00 (345.10, 603.55)	Z = −3.50	<.001
ALT (U/L), M (Q₁, Q₃)	15.63 (11.48, 22.40)	14.95 (10.67, 22.63)	Z = −0.63	0.532
AST (U/L), M (Q₁, Q₃)	31.61 (24.18, 41.25)	32.56 (25.71, 43.40)	Z = −0.74	0.457
Ferritin (ng/ml), M (Q₁, Q₃)	251.21 (191.82, 350.78)	222.27 (177.10, 281.02)	Z = −2.33	0.020
Fibrinogen (g/L), M (Q₁, Q₃)	3.82 (3.23, 4.38)	4.10 (3.33, 4.53)	Z = −0.69	0.491
D-dimer (mg/L), M (Q₁, Q₃)	0.68 (0.47, 0.93)	1.15 (0.67, 1.88)	Z = −3.76	<.001
CD4+/CD8+ Ratio, M (Q₁, Q₃)	1.50 (1.23, 1.83)	1.11 (0.88, 1.41)	Z = −4.98	<.001
IL-6 (pg/ml), M (Q₁, Q₃)	15.01 (4.76, 28.25)	30.40 (20.00, 51.05)	Z = -4.21	<.001

t: *t*-test, Z: Mann–Whitney test, χ^2^: Chi-square test.

SD, standard deviation, M, Median, Q₁, 1st Quartile, Q₃, 3st Quartile; WBC, white blood cell; N%, neutrophil percentage; PCT, procalcitonin; ESR, erythrocyte sedimentation rate; CRP, C-reactive protein; LDH, lactate dehydrogenase; ALT, alanine aminotransferase; AST, aspartate aminotransferase; IL-6, interleukin-6.

### 3.2 Variable selection using LASSO regression

**Figure 2:**
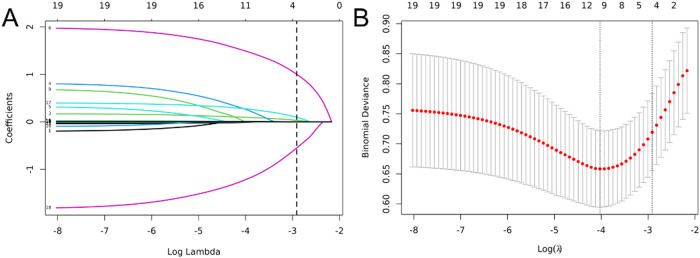
Variable selection using least absolute shrinkage and selection operator (LASSO) logistic regression. **(A)** LASSO coefficient profile of the 19 variables. With increasing penalties, coefficients of more variables are compressed to zero, ultimately selecting 4 variables with non-zero coefficients. **(B)** The optimal penalty coefficient lambda was selected using a tenfold cross-validation and minimization criterion. The binomial deviance curve was plotted vs. log(lambda), with dotted vertical lines drawn based on 1 standard error criterion. Four variables with non-zero coefficients were selected at the optimal lambda (*λ* = 0.054).

Nineteen variables, including sex, age, fever duration, presence of wet rales, wheezing, atelectasis, white blood cell (WBC) count, neutrophil percentage (N%), procalcitonin (PCT) level, erythrocyte sedimentation rate (ESR), C-reactive protein (CRP) level, lactate dehydrogenase (LDH) level, alanine aminotransferase (ALT) level, aspartate aminotransferase (AST) level, ferritin level, fibrinogen level, D-dimer level, CD4+/CD8 + ratio, and interleukin-6 (IL-6) level, were included in the LASSO regression to identify key predictors of PB in children with MPP (Figure 2A). As penalties increased, LASSO compressed the coefficients of most variables to zero, selecting those with non-zero coefficients (Figure 2A). The optimal penalty parameter (*λ*) was determined using tenfold cross-validation by minimizing the binomial deviance, resulting in the selection of four predictors: fever duration, presence of atelectasis, D-dimer levels, and CD4+/CD8 + ratio (Figure 2B). These selected variables represent the most influential factors in predicting PB risk, providing a robust foundation for the subsequent nomogram construction.To evaluate collinearity, variance inflation factors (VIFs) for the four predictors were calculated, all of which were less than 5, confirming the absence of multicollinearity.

### 3.3 Multivariable logistic regression analysis

The four predictors identified through LASSO regression—fever duration, presence of atelectasis, elevated D-dimer levels, and reduced CD4+/CD8 + ratio—were analyzed using multivariable logistic regression to determine their independent associations with PB in children with MPP. The Results are summarized in Table 2. The presence of atelectasis exhibited the strongest association with PB (*β* = 1.81, OR = 6.11, 95% CI 2.56–14.63, *P* < 0.001), followed by a reduced CD4+/CD8 + ratio (*β* = −1.78, OR = 0.17, 95% CI 0.06–0.50, *P* = 0.001). Fever duration (*β* = 0.17, OR = 1.19 per day, 95% CI 1.05–1.35, *P* = 0.007) and elevated D-dimer levels (*β* = 0.38, OR = 1.46 per mg/L, 95% CI 1.04–2.05, *P* = 0.030) were also significant predictors of PB. These findings confirm the independent predictive roles of these variables in PB development, highlighting their significance as key indicators for early identification and intervention strategies.

**Table 2:** Multivariable logistic regression for PB predictors in MPP children.

Variables	*β*	S.E	Z	*P*	OR (95%CI)
Atelectasis	1.81	0.44	4.07	<.001	6.11 (2.56–14.63)
Fever duration	0.17	0.06	2.71	0.007	1.19 (1.05–1.35)
D-dimer（mg/L）	0.38	0.17	2.17	0.030	1.46 (1.04–2.05)
CD4+/CD8+ Ratio	−1.78	0.55	−3.22	0.001	0.17 (0.06–0.50)

OR, odds ratio; CI, confidence interval.

### 3.4 Nomogram for PB risk prediction

**Figure 3:**
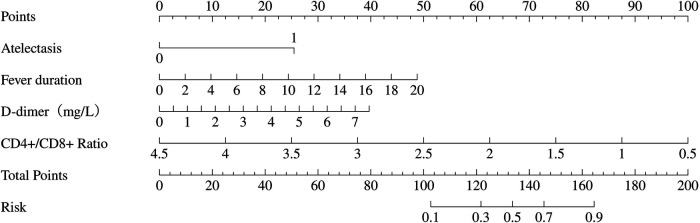
Nomogram to predict plastic bronchitis (PB) risk in children with *Mycoplasma pneumoniae* pneumonia (MPP). The nomogram is based on four independent predictors identified from multivariable logistic regression: fever duration, atelectasis, D-dimer, and CD4+/CD8 + ratio. To use the nomogram, mark the value of each predictor on its corresponding axis, draw a vertical line to the top “Points” axis to obtain the points, sum the points from all predictors, locate the total on the “Total Points” scale, and project it vertically to the “Risk” axis to determine the PB risk (0.1–0.9).

The variables identified in the multivariable logistic regression analysis including fever duration, presence of atelectasis, elevated D-dimer levels, and reduced CD4+/CD8 + ratio were used to construct a nomogram (Figure 3). Total points, based on the sum of points assigned to each predictor in the nomogram, are associated with the risk of PB, ranging from 0.1 to 0.9. This nomogram provides a visual and intuitive tool for clinicians to estimate individual patient risk of PB based on their specific clinical and laboratory profiles.

### 3.5 Nomogram performance evaluation

**Figure 4:**
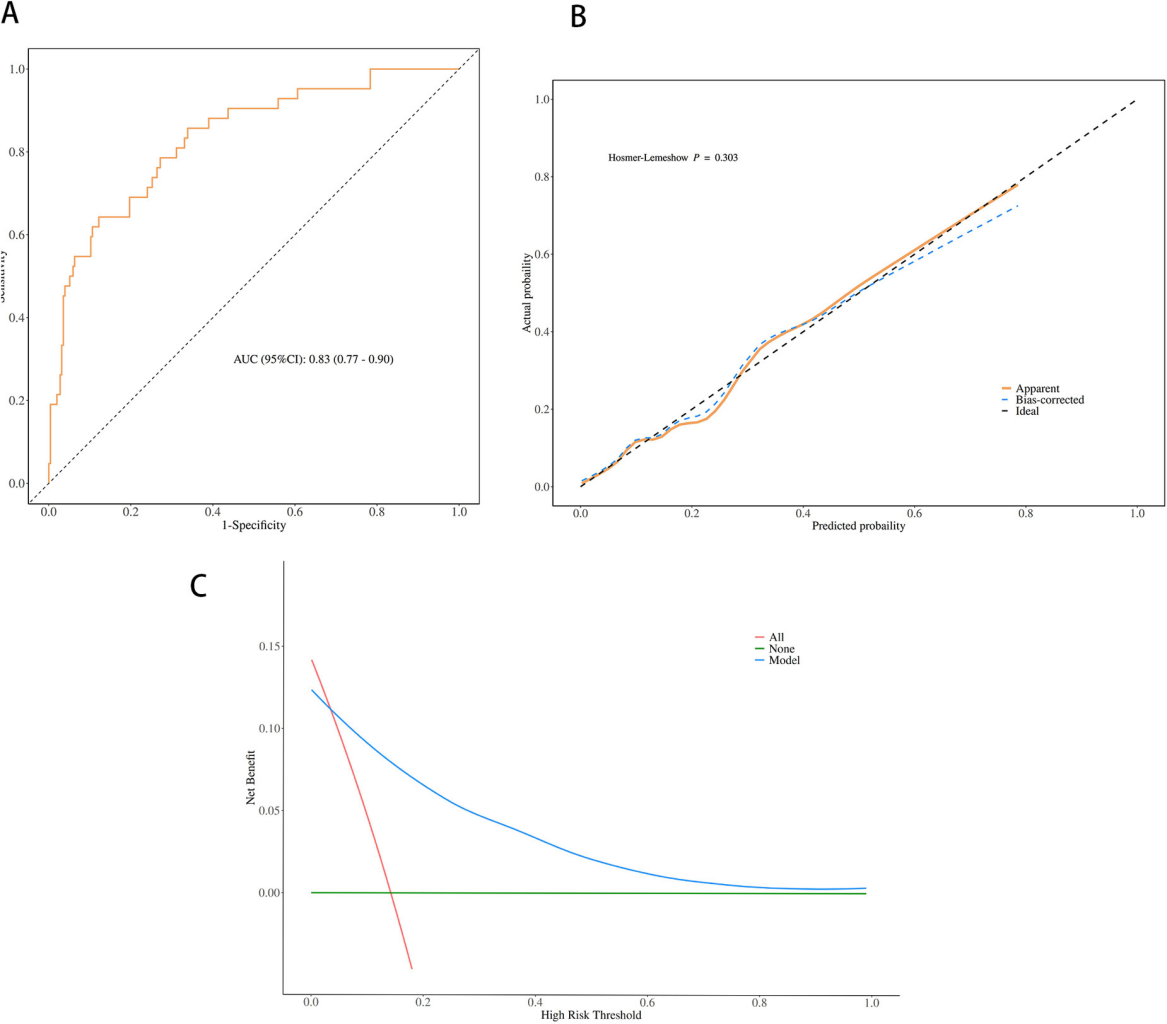
Performance evaluation of the nomogram for predicting plastic bronchitis (PB) risk in children with *Mycoplasma pneumoniae* pneumonia (MPP). **(A)** Receiver operating characteristic (ROC) curve; area under the curve (AUC) = 0.83 (95% CI 0.77–0.90). **(B)** Calibration curve; the Hosmer-Lemeshow test yielded a *p*-value of 0.303 (*P* > 0.05). **(C)** Decision curve analysis (DCA); the nomogram shows higher net benefit than “treat all” and “treat none” strategies across risk thresholds of 0.1 to 0.7.

The performance of the nomogram was evaluated for discrimination, calibration, and clinical utility (Figure 4), and it demonstrated good discriminatory performance with an area under the receiver operating characteristic (ROC) curve (AUC) of 0.83 (95% CI 0.77–0.90) (Figure 4A), good calibration with a Hosmer–Lemeshow test *p*-value of 0.303 (Figure 4B), and superior clinical utility compared to default strategies across risk thresholds of 0.1–0.7 (Figure 4C), demonstrating its potential to significantly improve early risk stratification and guide clinical decision-making.

### 3.6 Internal validation

**Figure 5:**
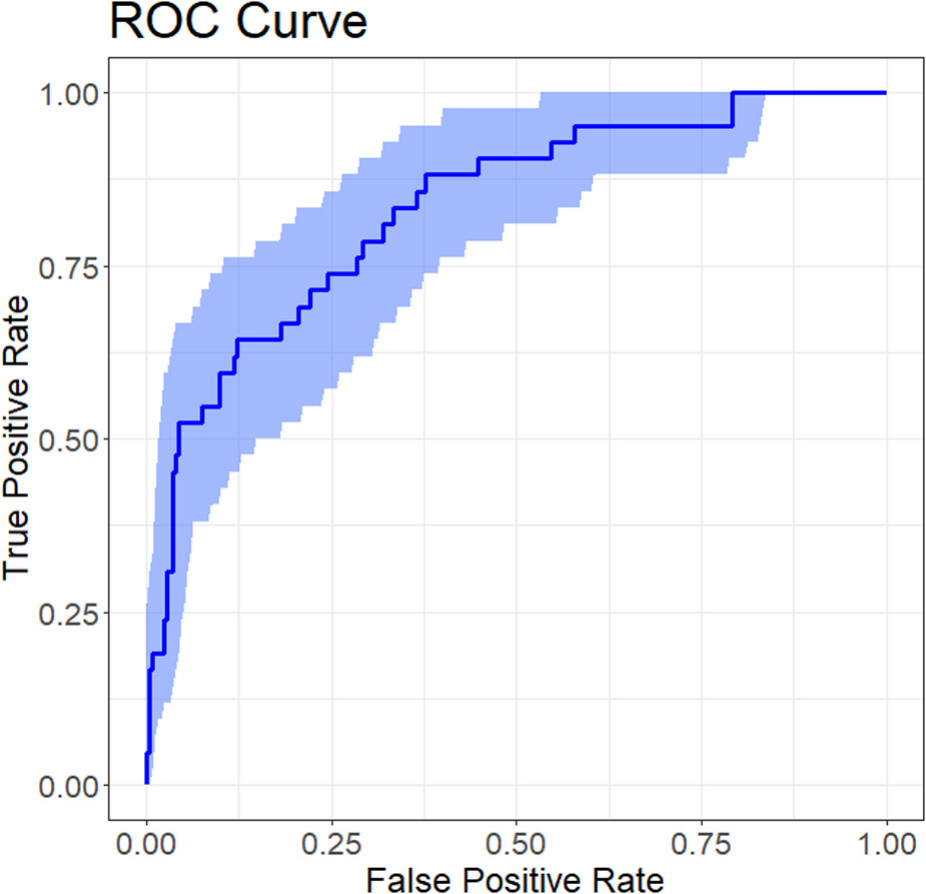
ROC curve for internal validation of the nomogram predicting plastic bronchitis (PB) risk in children with *Mycoplasma pneumoniae* pneumonia (MPP). The ROC curve illustrates the nomogram's discriminatory performance after internal validation using the Bootstrap method (1,000 resamples).

Internal validation of the nomogram was performed using the Bootstrap method with 1,000 resamples to assess the stability of the model's discriminatory performance. As shown in Figure 5, the C-index after Bootstrap validation was 0.834 (95% CI 0.767–0.899), consistent with the original AUC of 0.83, indicating good discriminatory ability with minimal overfitting, thus confirming the model's robustness and generalizability to similar patient populations.

## 4 Discussion

This study reports a plastic bronchitis (PB) incidence of 13.88% among 281 Mycoplasma pneumoniae pneumonia (MPP) patients, consistent with the 14.2% reported by Zhong et al. in a similar cohort requiring bronchoscopy (8). These findings highlight PB as a significant complication in severe MPP, necessitating early identification to guide timely bronchoscopic intervention and prevent outcomes like respiratory failure. Our nomogram, incorporating fever duration, atelectasis, D-dimer, and CD4+/CD8 + ratio, achieves robust discrimination (AUC = 0.83) and clinical utility (decision curve analysis, thresholds 0.1–0.7). By quantifying PB risk at admission, it enables clinicians to prioritize high-risk patients for bronchoscopy, potentially reducing complications such as airway obstruction or atelectasis progression. Unlike invasive diagnostic approaches, this nomogram leverages routine clinical and immunological markers, enhancing feasibility in pediatric settings. This tool addresses diagnostic delays in PB, reinforcing the study's rationale for advancing early detection and management in pediatric MPP.

Multivariate analysis identified four key predictors for the nomogram: fever duration, the presence of atelectasis, elevated D-dimer levels, and reduced CD4+/CD8 + ratio. The presence of atelectasis was the strongest predictor, reflecting its mechanistic link to cast-induced airway obstruction, a feature well-documented in severe respiratory infections (12). Atelectasis likely exacerbates airway obstruction by promoting mucus stasis and cast formation, creating a vicious cycle that worsens respiratory distress in patients with PB. Prolonged fever may reflect an ongoing inflammatory cascade, potentially driven by *Mycoplasma pneumoniae*'s ability to evade host immune responses, leading to sustained cytokine release and tissue damage that predisposes to the development of PB. Elevated D-dimer levels suggest a hypercoagulable state, aligning with coagulation abnormalities in severe MPP (13). This finding may indicate microvascular thrombosis or endothelial dysfunction in the airways, contributing to cast formation through fibrin deposition, a process often exacerbated by the inflammatory milieu in MPP (14). The reduced CD4+/CD8 + ratio, a novel predictor in this context, points to immune dysregulation, potentially driven by excessive cytotoxic T-cell activity, a hallmark of the immune response in MPP (15, 16). Jia et al. demonstrate that severe MPP (S-MPP) is characterized by increased Th1 cell frequencies and CD8+ T cells with an exhausted phenotype marked by elevated PD-1 expression (17). In our PB cohort, this decreased ratio may reflect an increased CD8+ T-cell proportion, impairing pathogen clearance and sustaining Th1-driven inflammation. Elevated IL-6 levels in our PB group support this inflammatory response but are not an independent predictor (18). Combined with increased D-dimer levels, consistent with S-MPP findings (17), this suggests that Th1-mediated inflammation promotes fibrin deposition and airway obstruction, critical for bronchial cast formation in PB. Thus, the reduced CD4+/CD8 + ratio serves as a key immunological marker linking persistent inflammation to PB's coagulative pathology.

The univariate analysis revealed elevated lactate dehydrogenase (LDH) and interleukin-6 (IL-6) levels in the PB group. These findings are consistent with the known roles of LDH as a marker of tissue injury and cell death, reflecting the extent of lung damage and inflammation in PB patients. Similarly, IL-6, a key pro-inflammatory cytokine, plays a central role in driving systemic inflammation and immune responses, contributing to the formation of bronchial casts and airway obstruction in PB. While these indicators were not identified as independent predictors in our multivariate model, likely due to their overlap with other significant factors, their elevation underscores the critical involvement of both direct tissue damage and systemic inflammatory processes in PB pathogenesis (19). Li et al. emphasized the role of C-reactive protein (CRP) and drug resistance in PB, highlighting heightened inflammation and treatment challenges posed by resistant strains (20). The lower white blood cell (WBC) count in PB cases (*P* = 0.061) mirrored variability seen in severe MPP, but did not reach statistical significance, suggesting no significant difference between the groups in this study (21). Our nomogram demonstrated strong performance and good calibration, with minimal overfitting confirmed by internal validation. The decision curve analysis indicated superior net benefit across a 0.1–0.7 risk threshold compared to uniform intervention strategies. Compared to Zhang et al.'s RMPP nomogram and Li et al.'s decision tree, this model enhances practicality by utilizing fewer variables (14, 22).

Clinically, the nomogram facilitates early PB detection using routine admission data, guiding targeted bronchoscopy, which is critical as PB prevalence increases among school-aged children with robust immune responses (23). Zhong et al. reported prolonged hospital stays in PB cases (12 vs. 7 days), a burden this tool could reduce through timely intervention (8). The role of the CD4+/CD8 + ratio suggests potential for immunomodulatory therapies, such as corticosteroids, though their efficacy needs further study (24). Zhang et al. linked PB to sequelae like bronchiolitis obliterans, underscoring the need for early intervention to improve long-term outcomes (22). By leveraging accessible markers, the nomogram supports resource-efficient management across diverse healthcare settings, aligning with actionable infectious disease strategies.

Our study possesses several key strengths. Firstly, it introduces the CD4+/CD8 + ratio as a novel immunological marker for predicting PB risk in MPP, addressing a critical gap in existing predictive models that often overlook immune dysregulation. Secondly, we employed robust statistical methodologies, including LASSO regression for rigorous variable selection, which minimizes overfitting and enhances model parsimony. Furthermore, the comprehensive evaluation of model performance through discrimination (AUC), calibration (Hosmer-Lemeshow test), and clinical utility (Decision Curve Analysis) provides a thorough assessment of the nomogram's predictive capabilities. The internal validation using Bootstrap resampling further confirms the model's robustness and generalizability within similar patient populations. These methodological strengths contribute to the reliability and clinical applicability of our nomogram, offering a practical tool for early risk stratification in pediatric MPP patients requiring bronchoscopy.

This study had some limitations. Firstly, the retrospective single-center design may introduce bronchoscopy-related selection bias. Secondly, the modest PB cohort of 39 cases (13.88%) may limit the model's generalizability and statistical power. Thirdly, the absence of longitudinal data to clarify the mechanistic role of the CD4+/CD8 + ratio requires cautious interpretation. The small PB cohort may also affect the model's stability, particularly for rare outcomes like PB, necessitating validation in larger cohorts. Future research prioritizing multicenter validation, longitudinal immune profiling to investigate T-cell dynamics, such as flow cytometry to assess T-cell subsets, and assess resistance patterns, such as A2063G mutation, using genomic sequencing to refine PB pathogenesis models is recommended.

In conclusion, this study identified fever duration, presence of atelectasis, elevated D-dimer levels, and reduced CD4+/CD8 + ratio as predictors of PB in MPP, developing a nomogram that enhances early risk stratification. This tool provides a practical approach to guide bronchoscopy and optimize outcomes in pediatric MPP, requiring further validation to advance infectious disease management.

## Data Availability

The original contributions presented in the study are included in the article/Supplementary Material, further inquiries can be directed to the corresponding author.
